# Generalized Born Implicit Solvent Models Do Not Reproduce Secondary Structures of *De Novo* Designed Glu/Lys Peptides

**DOI:** 10.1021/acs.jctc.1c01172

**Published:** 2022-06-10

**Authors:** Eric J. M. Lang, Emily G. Baker, Derek N. Woolfson, Adrian J. Mulholland

**Affiliations:** † Centre for Computational Chemistry, School of Chemistry, University of Bristol, Cantock’s Close, Bristol BS8 1TS, U.K.; ‡ School of Chemistry, University of Bristol, Cantock’s Close, Bristol BS8 1TS, U.K.; § BrisSynBio, University of Bristol, Life Sciences Building, Tyndall Avenue, Bristol BS8 1TQ, U.K.; ∥ School of Biochemistry, University of Bristol, Medical Sciences Building, University Walk, Bristol BS8 1TD, U.K.

## Abstract

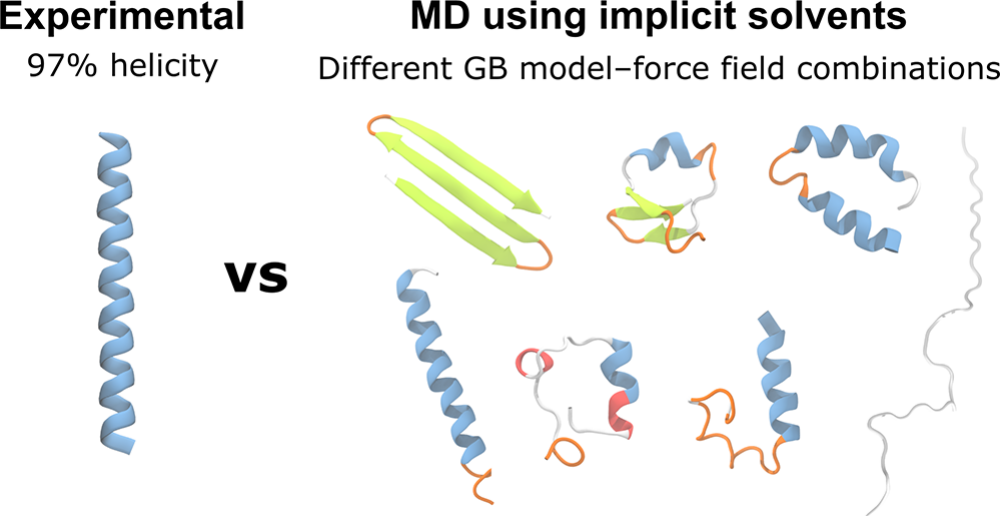

We test a range of standard generalized Born (GB) models and protein force fields for a set of five experimentally characterized, designed peptides comprising alternating blocks of glutamate and lysine, which have been shown to differ significantly in α-helical content. Sixty-five combinations of force fields and GB models are evaluated in >800 μs of molecular dynamics simulations. GB models generally do not reproduce the experimentally observed α-helical content, and none perform well for all five peptides. These results illustrate that these models are not usefully predictive in this context. These peptides provide a useful test set for simulation methods.

Molecular dynamics (MD) simulations have demonstrated their worth and are now an established technique, complementing experiment, in structural biology.^1^ MD simulations are also contributing increasingly to *de novo* protein design and also have a role in protein structure prediction. In design, typically many model structures must be evaluated, making MD simulations computationally expensive. MD simulations of designed proteins are often currently limited to short backbone-restrained simulations to improve side-chain packing.^2,3^ Protein design protocols could potentially benefit from incorporating more extensive unrestrained MD simulations routinely to test the stabilities of designed structures, to help design dynamical/conformational properties, and to improve understanding of sequence-to-structure/function relationships.^4−6^ An attractive, practical solution is to use implicit solvent models. These typically aim to include dielectric shielding and other effects of aqueous solvation, while avoiding the computational cost of explicit representation of large numbers of water molecules.^7,8^

Although explicit solvent simulations are in general more accurate, currently they are too computationally expensive for routine use in protein design pipelines that generate many constructs. Simulations using implicit solvent—such as those based on generalized Born (GB) models—are faster and easier to set up and analyze. Moreover, because protein dynamics are not damped by solvent viscosity, conformational space sampling is accelerated.^9,10^ Although GB models have been used successfully in many studies, they have well-known limitations: e.g., different GB model–force field combinations can to lead to very different results, with many combinations unable to reproduce native folds.^11−13^ Nonetheless, recent work suggests that the latest GB models and force fields have improved accuracy and reproduce the observed structures for a test set of small proteins, without the need to include nonelectrostatic terms such as effects of solvent accessible surface area.^14^

To test GB models, we simulate here a systematic series of *de novo* peptides, which we designed and experimentally characterized previously to explore electrostatic interactions in single α helices.^15^ They comprise blocks of negatively charged glutamate (Glu, E) and positively charged lysine (Lys, K) residues in sequences of the type (E_*x*_K_*x*_)_*n*_ or (K_*x*_E_*x*_)_*n*_, Table 1. Rational changes in lengths and sequences of the charged blocks result in strikingly different experimentally observed α-helical contents. Our aim was to identify a useful simulation model to assist protein design.

**Table 1:**
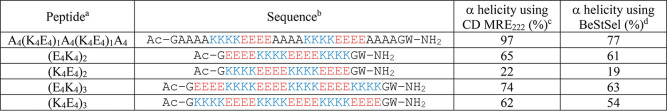
*De Novo* Peptides^a^ Used for Testing Different Force Field–GB Model Combinations

a Previously experimentally characterized by Baker et al.^15^

b Peptide sequences *N-* and *C*-terminally capped with acetyl (Ac) and amide (NH_2_) groups, respectively. Lys residues are colored blue, and Glu residues are colored red.

c Experimentally determined α helicities from circular dichroism (CD) spectroscopy measurements (5 °C in phosphate-buffered saline, pH 7.4) of mean residue ellipticity (MRE) at 222 nm.^15^ α Helicities were determined by BeStSel^24^ analysis of the CD spectra in the range of 200–250 nm.

Interest in such peptides is not limited to *de novo* design: they are also models for naturally occurring ER/K motifs, i.e., alternating repeats of Glu and Lys or arginine (Arg, R).^16^ Such motifs are found in all kingdoms of life and in ≈0.2–0.5% of all proteins and form stable α-helical structures in the absence of tertiary interactions.^17−19^ As such, they are also called single α-helical domains (SAHs). Notably, MD simulations of these domains using different force fields and with explicit TIP3P water models have been able to reproduce the experimental helicities.^16,20−22^

Here, we simulate five *de novo* sequences (Table 1) using five different GB models and 13 different force fields, using the AMBER16 biomolecular simulation program.^23^

We tested the five GB models available in AMBER16: the Hawkins, Cramer, Truhlar model^25^ (igb1); the Onufriev, Bashford, Case model^26,27^ (igb2) and its modified version^27^ (igb5); and the GBn model by Mongan, Simmerling, McCammon, Case, and Onufriev^28^ (igb7) and its modified version by Nguyen, Roe, and Simmerling^29^ (igb8). In combination with these, 13 AMBER force fields were tested: ff94,^30^ ff96,^31^ ff98,^32^ ff99,^33^ ff99SB,^34^ ff99SBildn,^35^ ff99SBnmr,^36^ ff03.r1,^37^ ff14SB,^38^ ff14SBonlysc,^38^ ff14ipq,^39^ fb15,^40^ and ff15ipq,^41^ i.e., 65 GB model/force field combinations in total.

The choice of GB models and AMBER was made for the following reasons. First, these GB models have been implemented in most of the major biomolecular MD packages: all five in AMBER and OpenMM; igb1, igb2, and igb5 in GROMACS; and igb2 in NAMD. Second, the GPU MD engine pmemd.cuda^42^ within AMBER is fast and ideal for rapid testing of multiple GB model–force field combinations.

Conditions from the experiments^15^ were replicated as closely as possible: the peptides were *N*-terminally acetylated and *C-*terminally amidated; the simulations were run at 278.15 K; and the ionic concentration was set to 0.137 mol/L to mimic that of phosphate buffered saline (pH 7.4). Glu and Lys side chains were treated as fully ionized, i.e., negatively and positively charged, respectively, yielding neutral peptides. Initially, single 6 μs simulations were run for each GB model–force field combination, with some systems repeated four times to test reproducibility (see below). All simulations used the same minimization, heating, and equilibration protocols (see Methods in the Supporting Information), starting from a fully α-helical structure of each peptide. The first 250 ns were discarded as further equilibration, yielding 5.75 μs of production MD for each. 5750 frames (saved every 1 ns) were analyzed for each peptide.

We began with peptide A_4_(K_4_E_4_)_1_A_4_(K_4_E_4_)_1_A_4_ because this is the most helical experimentally (Table 1). The helicities and structures from the MD simulations of this peptide for each GB model–force field combination are shown in Figure 1.

**Figure 1:**
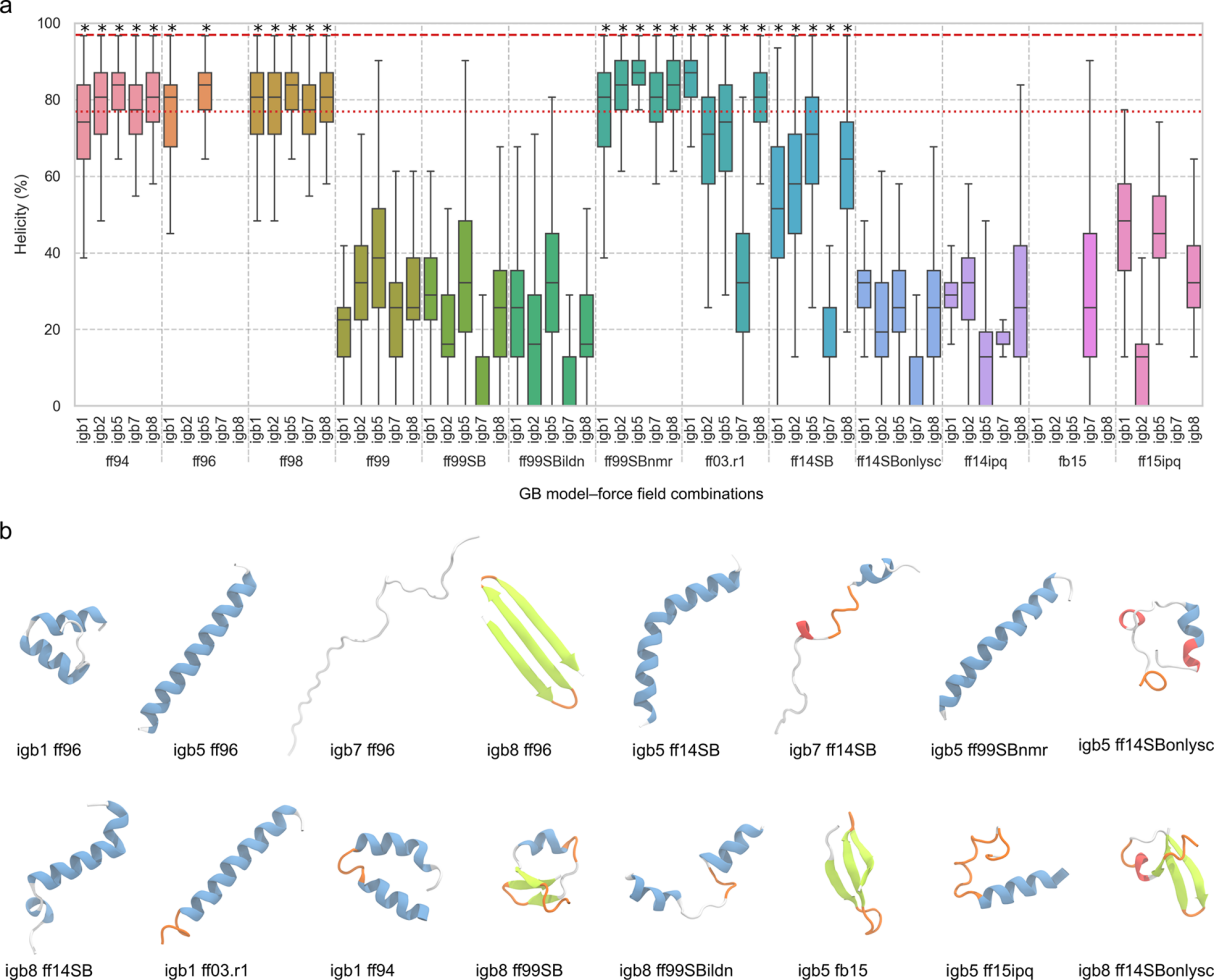
Predicted α helicities and final structures from MD simulations of A_4_(K_4_E_4_)_1_A_4_(K_4_E_4_)_1_A_4_ using 65 GB model–force field combinations. (**a**) Percentage helicity of A_4_(K_4_E_4_)_1_A_4_(K_4_E_4_)_1_A_4_ calculated with DSSP^43^ for the MD trajectories generated for all 65 combinations. The results are presented as boxplots with the boxes indicating the first quartile, the median, and the third quartile of the sample. The whiskers indicate 1.5 times the interquartile range. Gaps correspond to combinations for which the percentage α helicity is negligible. For each combination, 5750 frames were analyzed. Each force field is represented with a different color. The red dashed line represents the experimental helicity using mean residue ellipticity (MRE) at 222 nm,^15^ while the red dotted line corresponds to the helicity obtained with BeStSel by analyzing the CD spectra in the range of 200–250 nm. Asterisks indicate the GB model–force field combinations selected for further testing (Figure 2). (**b**) Backbone structures of A_4_(K_4_E_4_)_1_A_4_(K_4_E_4_)_1_A_4_ from selected GB model–force field combinations after 6 μs of MD. The peptides are colored by structure: α helix, blue; extended β-strand and β-bridge, green; π-helix, red; 3_10_ helix, purple; turn, orange; coil, white.

None of the GB model–force field combinations gave fully helical structures throughout the trajectories (Figure 1a). Moreover, a disconcertingly large array of conformations was observed (Figure 1b) with some structures being completely unfolded and others that did not maintain a stable secondary structure for more than a few nanoseconds or were completely reconfigured into β sheets (Movies S1 and S2). There is no experimental evidence (from circular dichroism or NMR spectroscopy) that the A_4_(K_4_E_4_)_1_A_4_(K_4_E_4_)_1_A_4_ peptide accesses these states in these conditions.^15^

The best performing combination was igb5 with ff99SBnmr (Movie S1), with a median helicity of ≈87% and an interquartile range of less than 10% over the course of the simulation. The slight loss of helicity was due to the unfolding of the terminal residues at both ends. Irrespective of the implicit solvent model, ff99SBnmr performed reasonably well for A_4_(K_4_E_4_)_1_A_4_(K_4_E_4_)_1_A_4_, in contrast to the related ff99SB, ff99SBildn, and ff99 force fields (median α helicity < 50%). The older force fields ff94 and ff98 yielded a median helicity above 75% and an interquartile range below 20%, regardless of the GB model used, and were among the best force fields at capturing the helical structure of this peptide.

The most recent GB model–force field combination, igb8 with ff14SBonlysc, which is recommended by the AMBER developers,^14,29^ did not maintain the starting α helix (Figure 1b, bottom right) or even a stable overall secondary structure (Movie S1). Changing the GB model did not significantly modify the outcome with this force field. Surprisingly, the ff14SB force field, which was parametrized for use with the explicit TIP3P water model, led to a higher simulated α helicity than ff14SBonlysc for each GB model, Figure 1 and Movie S1. Unsurprisingly, force fields ff14ipq, ff15ipq, and fb15, which were not designed to be compatible with GB models, were consistently poor at maintaining the peptide’s starting conformation (median α helicity < 50%) regardless of the GB model, Figures 1b and S2.

The simulations were sensitive to both the GB model and to the force field used: different predominant secondary structures were predicted by the same force field when different GB models were used and vice versa. Some force fields, e.g., ff96, ff03.r1, and ff14SB, were much more sensitive to the choice of the implicit solvent model, with some GB models giving a median helicity above 70% and others below 50%. For example, ff96 with igb5 captured the α helicity relatively well (median helicity ≈ 83%), but the same force field with igb8 predicted formation of a β hairpin (within 1 μs) which developed into a stable three-stranded sheet over the remainder of the simulation. In contrast, these force fields with igb7, the A_4_(K_4_E_4_)_1_A_4_(K_4_E_4_)_1_A_4_ showed the peptide as disordered (Figures 1b and S1 and Movie S2).

To test the reproducibility of these simulations, we ran a total of four replicas for each of several GB model–force field combinations (Figures S3 and S4). These repeated simulations gave similar structures, and the differences between replicas are, in most cases, minimal. Reassuringly, the same major conformational changes were sampled in all four replica runs, albeit at different times, e.g., the α helix-to-β structure transitions observed with the igb8–ff96 combination. These results indicate that a single 6 μs simulation sufficiently captures the performance of a GB model–force field combination for peptides of this size, but replica simulations should be run to ensure the reproducibility of the results for the best performing combinations.

Although none of the GB model–force field combinations maintained the experimentally observed helicity of A_4_(K_4_E_4_)_1_A_4_(K_4_E_4_)_1_A_4_, several gave a high degree of helicity (median helicity > 75%). Therefore, we tested these combinations, along with some others for comparison, on other peptides with lower experimental α-helical content, i.e., the pair (E_4_K_4_)_2_ (65% α helix) and (K_4_E_4_)_2_ (22% α helix), Table 1, which differ only in the order of the Glu_4_ and Lys_4_ blocks. For these peptides, 27 implicit solvent–force field combinations were tested, starting from fully α-helical conformations of both peptides and using the same protocols as for A_4_(K_4_E_4_)_1_A_4_(K_4_E_4_)_1_A_4_. We note that a peptide similar to (E_4_K_4_)_2_, differing only in the absence of Gly and of Gly and Trp residues at the *N* and *C* termini, respectively, has been studied by replica-exchange MD (REMD) in explicit TIP3P water using the ff03.r1 force field,^16^ which reproduced the experimentally determined helicity of the peptide at different temperatures with good accuracy.

In our simulations, the older force fields (ff94, ff96, ff98, and ff99SBnmr), which captured the high α-helical content of A_4_(K_4_E_4_)_1_A_4_(K_4_E_4_)_1_A_4_, consistently predicted high α helicities for both (E_4_K_4_)_2_ and (K_4_E_4_)_2_, Figure 2a. They did not discriminate between (E_4_K_4_)_2_ and (K_4_E_4_)_2_, regardless of the GB model, with median helicities for both peptides of ≈80% versus experimental values of 67% and 22%, respectively. These force fields appear to systematically favor and overestimate the α-helical structure, at least for these Glu/Lys-rich peptides. In contrast, the more recent force fields ff03.r1 and ff14SB gave lower α helicities that were closer to the experimentally determined values (except in the case of ff03.r1 with igb8), Figure 2a. Moreover, they correctly predicted (E_4_K_4_)_2_ to be more helical than (K_4_E_4_)_2_, although the differences in predicted helicities between the two peptides were less than measured experimentally. ff14SB performed better than ff03.r1. The combination of ff14SB with igb7, which failed to predict the experimentally determined α helicity of A_4_(K_4_E_4_)_1_A_4_(K_4_E_4_)_1_A_4_ (13% vs 97%) (Figure 1), gave the best prediction of the difference in helicity between (E_4_K_4_)_2_ and (K_4_E_4_)_2_ (63% and 21%, respectively). Overall, our results reveal that even when a particular GB model–force field combination describes a particular peptide well, it may fail for a closely related homologue, for instance, one with the same composition but a different order of the amino acid residues.

**Figure 2:**
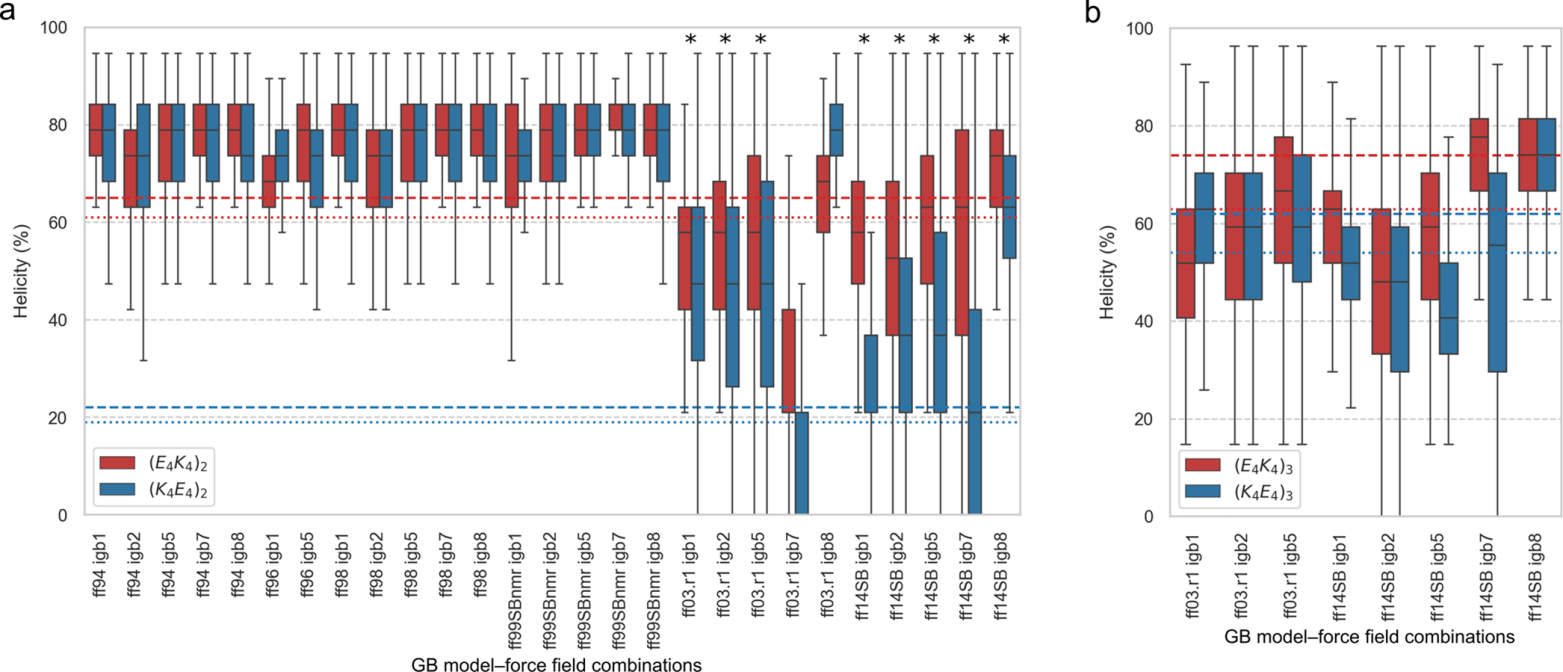
DSSP^43^ calculated α helicities of (E_4_K_4_)_*n*_ and (K_4_E_4_)_*n*_ peptides, where *n* = 2, 3. (**a**) Percentage α helicities of (E_4_K_4_)_2_ (red) and (K_4_E_4_)_2_ (blue) for the MD trajectories generated from 27 GB model–force field combinations. Asterisks indicate the combinations selected for MD simulations of longer peptide variants (E_4_K_4_)_3_ and (K_4_E_4_)_3_. (**b**) Percentage α helicities of (E_4_K_4_)_3_ (red) and (K_4_E_4_)_3_ (blue) for the MD trajectories from eight GB model–force field combinations. (**a** and **b**) The results are presented as boxplots as in Figure 1. The dashed lines show the experimentally measured helicities for each (E_4_K_4_)_*n*_ peptide in red and each (K_4_E_4_)_*n*_ peptide in blue using mean residue ellipticity (MRE) at 222 nm,^15^ and the dotted lines correspond to the helicity obtained with BeStSel by analyzing the CD spectra in the range of 200–250 nm.

To test this last point further, we took the eight GB model–force field combinations that performed best for (E_4_K_4_)_2_ and (K_4_E_4_)_2_ and modeled their longer counterparts, namely (E_4_K_4_)_3_ and (K_4_E_4_)_3_, Table 1, which have a smaller difference in experimental α helicities, i.e., 74% and 62%, respectively. Again, the different methods predicted a wide range of helicities (Figure 2b). Both ff03.r1 with igb5 and ff14SB with igb7 gave results in reasonable agreement with the experimental helicity for the two peptides, with median α helicities of 67% and 78%, respectively, for (E_4_K_4_)_3_ and 59% and 56%, respectively, for (K_4_E_4_)_3_. ff14SB with igb1 and igb5 also correctly predicted (E_4_K_4_)_3_ as the more helical peptide, although the median helicities were 10% to 20% lower than experiment.

Overall, the combination of ff14SB with igb7 stood out, having predicted the percentage helicities for (E_4_K_4_)_2_, (K_4_E_4_)_2_, (E_4_K_4_)_3_, and (K_4_E_4_)_3_ reasonably well; i.e., all were within 10% of the experimental values (Figure 2). However, it failed to predict the high helicity of A_4_(K_4_E_4_)_1_A_4_(K_4_E_4_)_1_A_4_, Figure 1a, returning random conformations instead (Figure 1b). Moreover, the combinations that best predicted the helicity of A_4_(K_4_E_4_)_1_A_4_(K_4_E_4_)_1_A_4_ did not replicate the percentage helicities for the other four peptides and almost always failed to predict the more helical of the paired designs. Thus, none of the GB–force field combinations tested here are a reliable predictor for all five of the peptides that we tested.

To test these findings further, we performed further analysis of the original experimental CD data using a different method, BeStSel^24,44^ (see Supplementary Results), which fits CD spectra to linear combinations of components derived from DSSP. Fitting of the experimental CD data was performed using the BeStSel Web server^24^ for the 200–250 nm region. BeStSel gave some differences in values of α helicity from those originally reported;^15^ in particular for A_4_(K_4_E_4_)_1_A_4_(K_4_E_4_)_1_A_4_, which was calculated to be 77% α helical using BeStSel vs 97% originally (Table 1). However, the results obtained from using BeStSel-predicted helicities did not change our conclusions: none of the GB model–force field combinations were predictive for all five peptides, and none were able to correctly model the fraction of other secondary structures identified by BeStSel.

Several important factors should be noted. REMD^45^ was not used here,because the goal was to look at a fast approach that can be applied in protein design. REMD might improve convergence but is not likely to change the overall conclusion that the GB models are not predictive of secondary structures adopted by these peptides: as shown above, conformational sampling of these peptides on these time scales under these conditions appears reasonable for these purposes. Similarly, the surface area (SA) term, which approximates nonpolar contributions to the solvation free energy, was not computed because the goal was to test fast approaches useful to nonexperts (i.e., using default options), and at the time the simulations were conducted, GBSA simulations could not be run on GPUs with AMBER. Although approximating nonpolar solvation using solvent accessible surface area has limitations,^46,47^ repeating the simulations using GBSA would be interesting. However, it is unlikely to change the results drastically as the electrostatic term is expected to be dominant for these peptides. Neglecting nonpolar terms has been shown to perform well for small peptides.^14,29^ Also, all the Glu and Lys residues were treated as ionized. Interactions between such charged residues can lead to changes in effective p*K*_a_ (coupled p*K*_a_s) and hence in the protonation state.^48^ Using constant pH, MD^49,50^ could shed further light on this and on any effect on the secondary structure. Finally, it would be interesting to explore combinations of force fields with explicit solvent models on the same Glu- and Lys-rich peptides.

In summary, none of the GB model–force field combinations that we have tested accurately reproduce the experimentally measured α helicities of the five peptides. While some GB model–force field combinations systematically show a high degree of helicity, irrespective of the peptide, others are only predictive for peptides with high or intermediate α helicities. Furthermore, some combinations predict entirely incorrect conformations, including β-rich or disordered structures, for which there is no experimental evidence. Therefore, our simulations serve as a warning of the potential unreliability of GB models for some predictions of protein/peptide properties and a reminder of the importance of the force field: for the peptides studied here, changing the force field for a given GB model usually leads to changes that are more marked than changing the GB model for a given force field. They also highlight the importance, whenever possible, of rigorously testing GB models and force fields against experimental data.

Explicit solvent simulations give good results for similar peptides.^16,20−22^ The recommendation to the protein designer currently would therefore be to use explicit solvent MD simulations. Inclusion of nonelectrostatic solvation terms may improve results and would be recommended for protein design applications with implicit solvent models. The small peptides here may not be representative of the behavior of larger, folded proteins, and implicit solvent simulations may well be useful for refining such structures, which are likely to remain folded on reasonable time scales. We also acknowledge the limited sequence variation of the highly repetitive peptides modeled, as well as their relatively unusual charge distributions, which may make them particularly challenging to model. However, similar features are found in naturally occurring proteins.^16−19^ These *de novo* peptides provide a useful training set for simulations and machine learning and for testing solvent models and protein force fields.

## Supporting Information Available

Methods; supplementary results; Figure S1, percentage α helicity of A_4_(K_4_E_4_)_1_A_4_(K_4_E_4_)_1_A_4_ as function of simulation time; Figure S2, average secondary structures; Figure S3, predicted α helicity from replica MD simulations; Figure S4, α helicity as function of time from replica MD simulations; Figure S5, percentage α helicity of (E_4_K_4_)_2_ and (K_4_E_4_)_2_ peptides; Figure S6, percentage helicity of (E_4_K_4_)_3_ and (K_4_E_4_)_3_ peptides; Figures S7–S11, results of BeStSel fitting of experimental CD curve for A_4_(K_4_E_4_)_1_A_4_(K_4_E_4_)_1_A_4_, (E_4_K_4_)_2_, (K_4_E_4_)_2_, (E_4_K_4_)_3_, and (K_4_E_4_)_3_, respectively; and Table S1, fraction helicity calculated from experimental CD data using BeStSel (PDF)
Movie S1, trajectories of 6 μs MD simulations of A_4_(K_4_E_4_)_1_A_4_(K_4_E_4_)_1_A_4_ using igb5 (MP4)
Movie S2, trajectories of 6 μs MD simulations of A_4_(K_4_E_4_)_1_A_4_(K_4_E_4_)_1_A_4_ using ff96 (MP4)

## Notes

Data access statement: All structures, scripts and data used in this study are available at https://github.com/eric-jm-lang/GB_simulations_de_novo_peptides.

## Abbreviations

GB: generalized Born
MD: molecular dynamics
CD: circular dichroism
REMD: replica exchange molecular dynamics
