# Supplementary material for: Generalized Born Implicit Solvent Models Do Not Reproduce Secondary Structures of De Novo Designed Glu/Lys Peptides
Source: J Chem Theory Comput. 2022 Jun 10;18(7):4070–6. doi: 10.1021/acs.jctc.1c01172 (PMC9281390; doi:10.1021/acs.jctc.1c01172)
Supplement: Supplementary file 1 — ct1c01172_si_001.pdf [file ct1c01172_si_001.pdf]

Generalized Born implicit solvent models do not reproduce secondary structures of *de novo* designed Glu/Lys peptides

*Eric J. M. Lang[1,2,3 *], Emily G. Baker[2,3], Derek N. Woolfson[2,3,4], Adrian J. Mulholland[1,2*]*

[1]Centre for Computational Chemistry, School of Chemistry, University of Bristol, Cantock's Close, Bristol, BS8 1TS, UK. [2]School of Chemistry, University of Bristol, Cantock's Close, Bristol, BS8 1TS, UK [3]BrisSynBio, University of Bristol, Life Sciences Building, Tyndall Avenue, Bristol BS8 1TQ, UK.[4]School of Biochemistry, University of Bristol, Medical Sciences Building, University Walk, Bristol, BS8 1TD, UK.

[*]Corresponding authors: eric.lang@bristol.ac.uk, adrian.mulholland@bristol.ac.uk

METHODS 2

SUPPLEMENTARY RESULTS...4

SUPPLEMENTARY FIGURES 5

Figure S1 ... 7
Figure S2 ... 9
Figure S3 ... 9
Figure S4 ... 11
Figure S5 ... 13
Figure S6 ... 13
Figure S7 ... 14
Figure S8 ... 15
Figure S9 ... 16
Figure S10 ... 17
Figure S11 ... 18
SUPPLEMENTARY TABLE ... 19
Table S1 ... 19
SUPPLEMENTARY MOVIES ... 19
Movie S1 ... 19
Movie S2 ... 20
REFERENCES ... 20

METHODS

All the simulations were run using AMBER16[1] and were started from a fully α-helical models of the following peptides: $A_4(K_4E_4)_1A_4(K_4E_4)_1A_4$, $(E_4K_4)_3$, $(K_4E_4)_3$, $(E_4K_4)_2$ and $(K_4E_4)_2$. Each initial peptide model was built in Chimera[2] using the 'Build Structure' tool, selecting an α-helix

structure and the Dunbrack rotamer library[3]. The peptides were acetylated at the *N*-terminus residue and amidated at the *C*-terminus, yielding neutral peptides. The required AMBER topology and coordinate files were prepared using the tleap program in AMBER16.

Five generalized Born (GB) models were tested:

- The Hawkins, Cramer, Truhlar model[4] (igb1, GB^{HCT}), corresponding to the igb=1 option and used in combination with the set of atomic radii '*mbondi*'.
- The Onufriev, Bashford, Case model[5,6] (igb2, GB^{OBC}), corresponding to the igb=2 option and used in combination with the set of atomic radii '*mbondi2*'.
- The modified Onufriev, Bashford, Case model[6] (igb5, GB^{OBC2}), corresponding to the igb=5 option and used in combination with the set of atomic radii '*mbondi2*'.
- The GBn model[7] described by Mongan, Simmerling, McCammon, Case and Onufriev (igb7, GB^{Neck}) corresponding to the igb=7 option and used in combination with the set of atomic radii '*bondi*'.
- The modification of the GBn model[8] by Nguyen, Roe Simmerling (igb8, GB^{Neck2}), corresponding to the igb=8 option used in combination with the set of atomic radii '*mbondi3*'.

These GB models were tested in combination with 13 AMBER forcefields: ff94[9], ff96[10], ff98[11], ff99[12], ff99SB[13], ff99SBildn[14], ff99SBnmr[15], ff03.r1[16], ff14SB[17], ff14SBonlysc[17], ff14ipq[18], fb15[19] and ff15ipq[20].

In order to accelerate the MD simulations, the hydrogen mass repartitioning (HMR) method[21] was applied, enabling the use of a 4 fs timestep. All of the simulations were performed using the same protocol, starting with 1000 steps of energy minimization of the hydrogen atoms, followed by 1000 steps of minimization of the whole peptide. The peptides were then heated to 278.15 K (to match the temperature of the circular dichroism experiments used to determine the fraction of helicity[22]) over 200 ps using a Langevin thermostat and applying a restraint of 10 $kcal.mol^{-1}.Å^{-2}$ on all backbone atoms. The peptides were then equilibrated for 200 ps with a restraint of 1 $kcal.mol^{-1}.Å^{-2}$ on backbone atoms, followed by an additional 200 ps using a restraint of 0.1 $kcal.mol^{-1}.Å^{-2}$ on backbone atoms. Production MD was then run for 6 μs. From the heating phase onward, SHAKE was applied to all hydrogen bonds, the Langevin thermostat collision frequency was set to 1.0 ps^{-1}, an infinite cut-off was used, and the ionic concentration was set to 0.137 mol/L. Equilibration and production simulations were run on the GPUs using pmemd.cuda.[23] Nonpolar

solvation contributions approximated by surface area calculations were not computed in this work (gbsa=0, default in AMBER).

Following a preliminary analysis, the first 250 ns of each simulation were discarded as equilibration, giving a total of 5.75 μs of analyzed production MD for each simulation. The secondary structure content of the peptides for each trajectory was analyzed using the DSSP algorithm[24] implemented in CPPTRAJ[25].

SUPPLEMENTARY RESULTS

We reanalyzed the experimental CD data collected for the featured peptides using the BeStSel method[26,27] which fits CD spectra to linear combinations of components derived from DSSP. It should be noted that fit was only performed for the 200-250 nm region because fitting over the entire region for which data was collected (190-250 nm) led to very poor agreement with the experimental curve, this might be due to reduced resolution below 200 nm due to PBS absorbance, or fitting errors below 200 nm.

BeStSel gave some differences in values of α helicity from those originally reported; in particular for $A_4(K_4E_4)_1A_4(K_4E_4)_1A_4$, which was calculated to be 77% α helical using BeStSel vs.

97% originally, Table 1. However, the values obtained from BeStSel do not change our conclusions: taking a helicity of 77% instead of 97% for $A_4(K_4E_4)_1A_4(K_4E_4)_1A_4$, the same GB-forcefield combinations would still be selected. The most predictive combinations are: ff94 with igb1 (74%), igb2 (81%), igb7 (77%) and igb8 (81%); ff96 with igb1 (81%); ff98 with igb1 (81%), igb2 (81%), igb7 (77%) and igb8 (81%); ff99SBnmr with igb1 (81%) and igb7 (81%); ff03.r1 with igb5 (74%) and igb8 (81%). Although we note that the results from the MD simulations of $A_4(K_4E_4)_1A_4(K_4E_4)_1A_4$ agree better with the BeStSel predictions than with the originally reported α helicities, apart from ff03.r1, all of the best-performing combinations are biased toward high α-helical content (Figure 2). Moreover, none of the combinations correctly predict the fraction of the other secondary structures identified by BeStSel (Figure S2 and S7, Table S1): *i.e.* antiparallel β strand, 10%; turn, 3%; other (includes coil, bend, β bridge, π helix and 3_{10} helix), 10%. The BeStSel calculated α helicities for $(E_4K_4)_2$ and $(K_4E_4)_2$ at 61 and 19%, respectively, closely matched those originally reported. The best combinations were ff14SB with igb1 and igb7. For $(E_4K_4)_3$ and $(K_4E_4)_3$ (63 and 54%) ff14SB with igb1 was the best performing combination, which in contrast did not perform well when using the α helicities from Baker *et al.*

Overall, using a different approach to assess the experimental helicity did not improve the agreement with experiment: none of the GB model–forcefield combinations were predictive for all five peptides and none were able to correctly model the other secondary structures identified by BeStSel.

SUPPLEMENTARY FIGURES

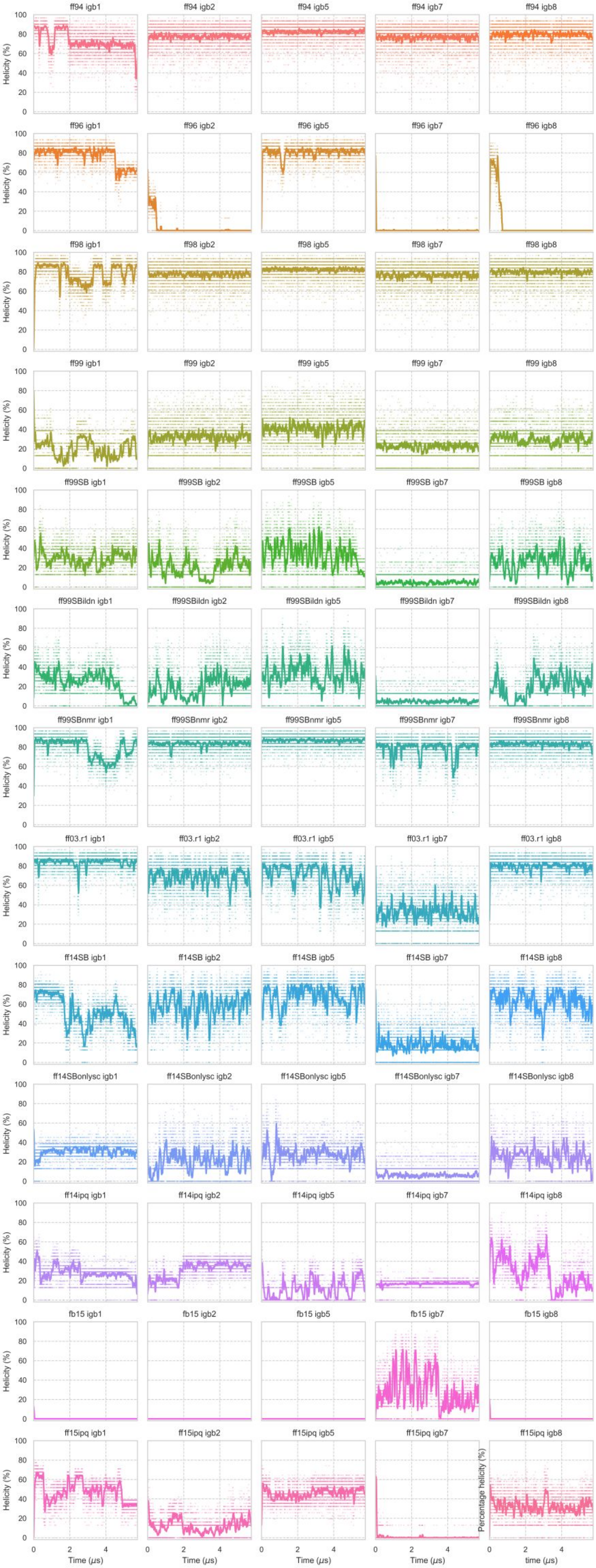
ff94 igb1
ff94 igb2
ff94 igb5
ff94 igb7
ff94 igb8
ff96 igb1
ff96 igb2
ff96 igb5
ff96 igb7
ff96 igb8
ff98 igb1
ff98 igb2
ff98 igb5
ff98 igb7
ff98 igb8
ff99 igb1
ff99 igb2
ff99 igb5
ff99 igb7
ff99 igb8
ff99SB igb1
ff99SB igb2
ff99SB igb5
ff99SB igb7
ff99SB igb8
ff99SBildn igb1
ff99SBildn igb2
ff99SBildn igb5
ff99SBildn igb7
ff99SBildn igb8
ff99SBnmr igb1
ff99SBnmr igb2
ff99SBnmr igb5
ff99SBnmr igb7
ff99SBnmr igb8
ff03.r1 igb1
ff03.r1 igb2
ff03.r1 igb5
ff03.r1 igb7
ff03.r1 igb8
ff14SB igb1
ff14SB igb2
ff14SB igb5
ff14SB igb7
ff14SB igb8
ff14SBonlysc igb1
ff14SBonlysc igb2
ff14SBonlysc igb5
ff14SBonlysc igb7
ff14SBonlysc igb8
ff14ipq igb1
ff14ipq igb2
ff14ipq igb5
ff14ipq igb7
ff14ipq igb8
fb15 igb1
fb15 igb2
fb15 igb5
fb15 igb7
fb15 igb8
ff15ipq igb1
ff15ipq igb2
ff15ipq igb5
ff15ipq igb7
ff15ipq igb8
Helicity (%)
Percentage helicity (%)
Time (μs)
time (μs)

Figure S1. Percentage α-helicity of $A_4(K_4E_4)_1A_4(K_4E_4)_1A_4$ as a function of simulation time. For each subplot, the dots represent the actual calculated values, and the solid line corresponds to the moving average over a window of 50 ns. Each row corresponds to a particular GB model–forcefield combination and each column represents a unique MD run. Note that the percentage helicity data is discrete as because of the finite number of residues constituting the peptide. For each combination a total of 5750 frames were analyzed. The plots are colored by GB model–forcefield combinations

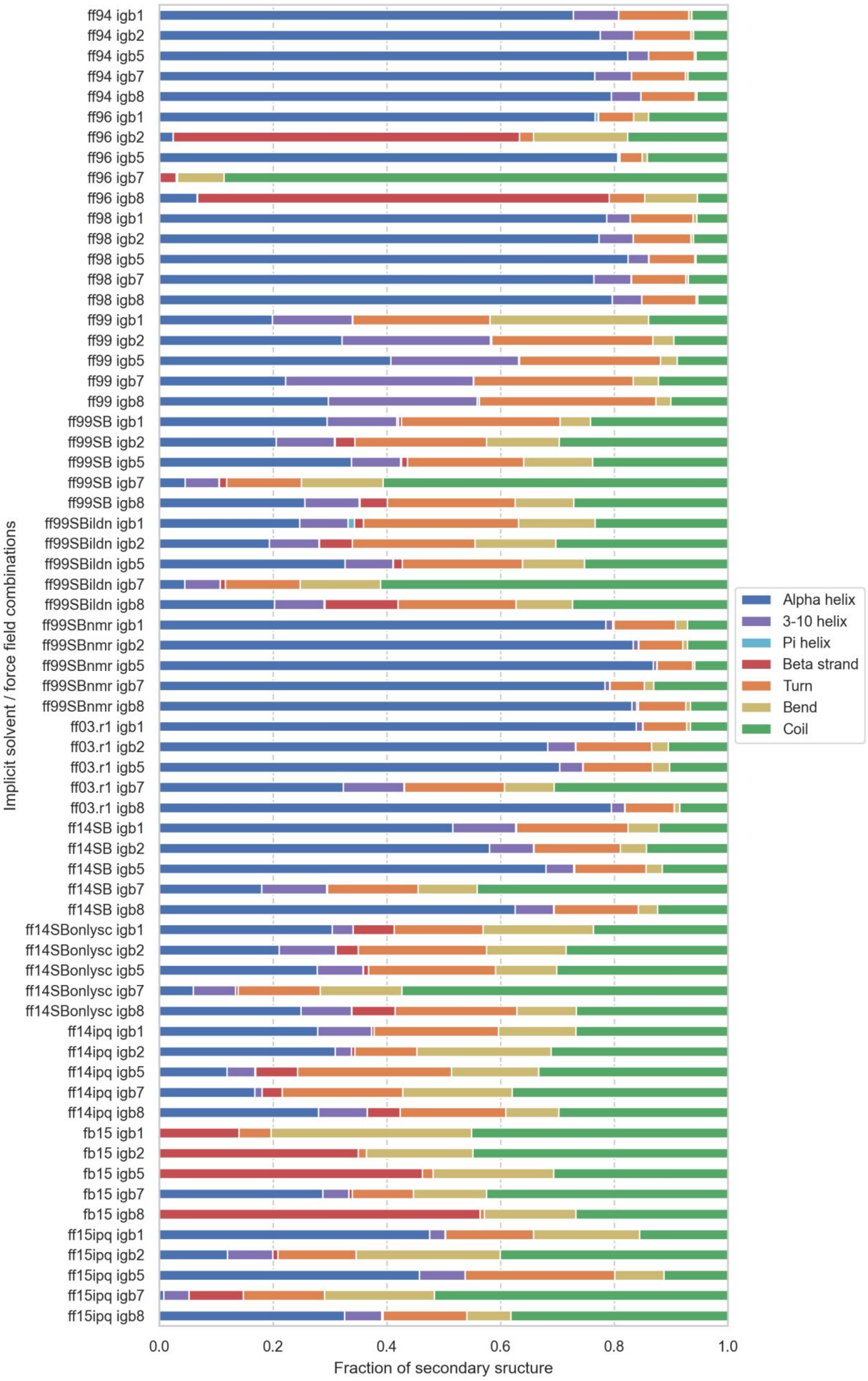

ff94 igb1
ff94 igb2
ff94 igb5
ff94 igb7
ff94 igb8
ff96 igb1
ff96 igb2
ff96 igb5
ff96 igb7
ff96 igb8
ff98 igb1
ff98 igb2
ff98 igb5
ff98 igb7
ff98 igb8
ff99 igb1
ff99 igb2
ff99 igb5
ff99 igb7
ff99 igb8
ff99SB igb1
ff99SB igb2
ff99SB igb5
ff99SB igb7
ff99SB igb8
ff99SBildn igb1
ff99SBildn igb2
ff99SBildn igb5
ff99SBildn igb7
ff99SBildn igb8
ff99SBnmr igb1
ff99SBnmr igb2
ff99SBnmr igb5
ff99SBnmr igb7
ff99SBnmr igb8
ff03.r1 igb1
ff03.r1 igb2
ff03.r1 igb5
ff03.r1 igb7
ff03.r1 igb8
ff14SB igb1
ff14SB igb2
ff14SB igb5
ff14SB igb7
ff14SB igb8
ff14SBonlysc igb1
ff14SBonlysc igb2
ff14SBonlysc igb5
ff14SBonlysc igb7
ff14SBonlysc igb8
ff14ipq igb1
ff14ipq igb2
ff14ipq igb5
ff14ipq igb7
ff14ipq igb8
fb15 igb1
fb15 igb2
fb15 igb5
fb15 igb7
fb15 igb8
ff15ipq igb1
ff15ipq igb2
ff15ipq igb5
ff15ipq igb7
ff15ipq igb8
Implicit solvent / force field combinations
0.0
0.2
0.4
0.6
0.8
1.0
Fraction of secondary sructure
Alpha helix
3-10 helix
Pi helix
Beta strand
Turn
Bend
Coil

Figure S2. Average secondary structures sampled over the course of the MD simulations for $A_4(K_4E_4)_1A_4(K_4E_4)_1A_4$ for each GB model–forcefield combination.

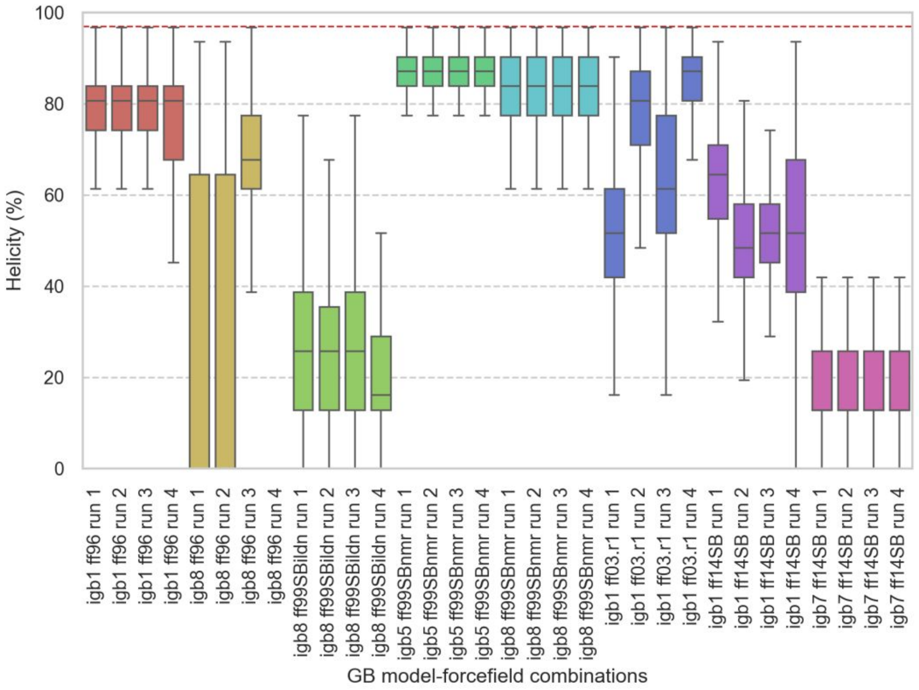

Figure S3. Predicted α-helicity from replica MD simulations for a selection of GB model–forcefield combinations for $A_4(K_4E_4)_1A_4(K_4E_4)_1A_4$. The results are presented as boxplots, with the boxes indicating the first quartile, the median and the third quartile of the sample. The whiskers indicate 1.5 times the interquartile range. Each forcefield–GB model combination is represented with a different color. The percentage α-helix was calculated with DSSP.

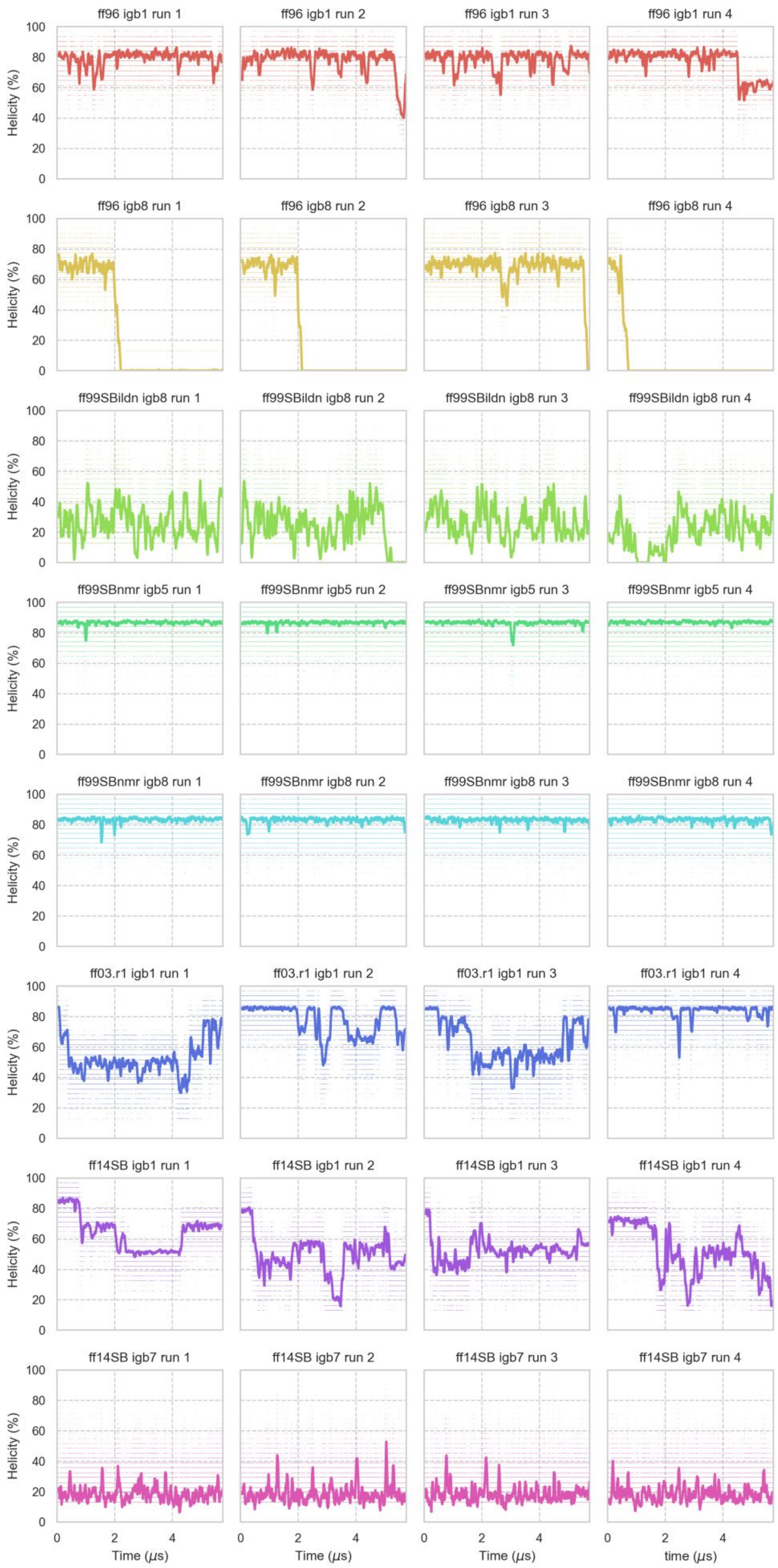
ff96 igb1 run 1
ff96 igb1 run 2
ff96 igb1 run 3
ff96 igb1 run 4
ff96 igb8 run 1
ff96 igb8 run 2
ff96 igb8 run 3
ff96 igb8 run 4
ff99SBildn igb8 run 1
ff99SBildn igb8 run 2
ff99SBildn igb8 run 3
ff99SBildn igb8 run 4
ff99SBnmr igb5 run 1
ff99SBnmr igb5 run 2
ff99SBnmr igb5 run 3
ff99SBnmr igb5 run 4
ff99SBnmr igb8 run 1
ff99SBnmr igb8 run 2
ff99SBnmr igb8 run 3
ff99SBnmr igb8 run 4
ff03.r1 igb1 run 1
ff03.r1 igb1 run 2
ff03.r1 igb1 run 3
ff03.r1 igb1 run 4
ff14SB igb1 run 1
ff14SB igb1 run 2
ff14SB igb1 run 3
ff14SB igb1 run 4
ff14SB igb7 run 1
ff14SB igb7 run 2
ff14SB igb7 run 3
ff14SB igb7 run 4
Helicity (%)
Time (μs)
time (μs)

Figure S4. α-helicity as a function of time from replica MD simulations for a selection of GB model–forcefield combinations for $A_4(K_4E_4)_1A_4(K_4E_4)_1A_4$. The results are presented as percentage helicity as a function of simulation time. For each subplot, the dots represent the actual calculated values, and the solid line corresponds to the moving average over a window of 50 ns. The plots are colored by GB model–forcefield combination.

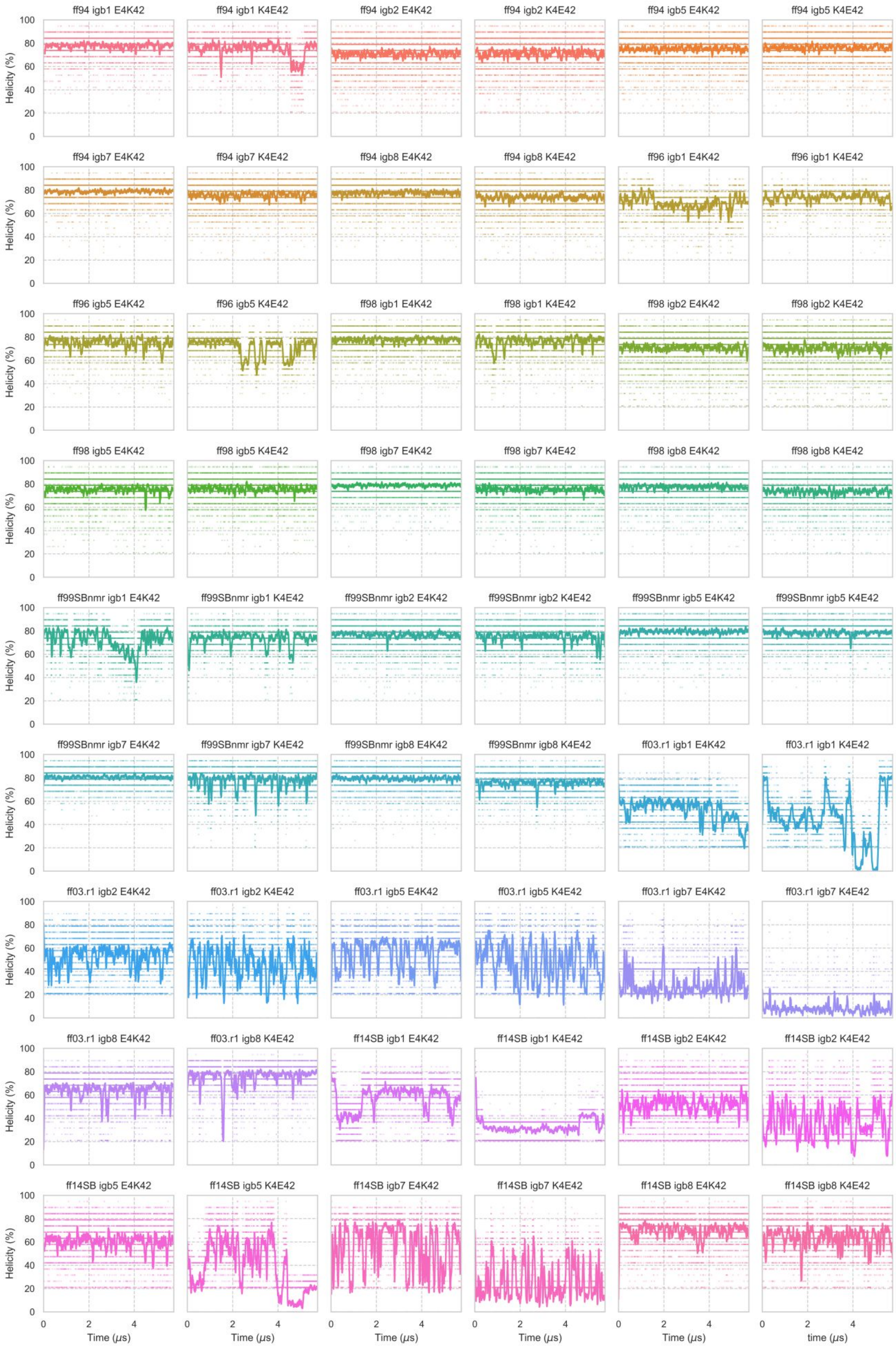
ff94 igb1 E4K42
ff94 igb1 K4E42
ff94 igb2 E4K42
ff94 igb2 K4E42
ff94 igb5 E4K42
ff94 igb5 K4E42
ff94 igb7 E4K42
ff94 igb7 K4E42
ff94 igb8 E4K42
ff94 igb8 K4E42
ff96 igb1 E4K42
ff96 igb1 K4E42
ff96 igb5 E4K42
ff96 igb5 K4E42
ff98 igb1 E4K42
ff98 igb1 K4E42
ff98 igb2 E4K42
ff98 igb2 K4E42
ff98 igb5 E4K42
ff98 igb5 K4E42
ff98 igb7 E4K42
ff98 igb7 K4E42
ff98 igb8 E4K42
ff98 igb8 K4E42
ff99SBnmr igb1 E4K42
ff99SBnmr igb1 K4E42
ff99SBnmr igb2 E4K42
ff99SBnmr igb2 K4E42
ff99SBnmr igb5 E4K42
ff99SBnmr igb5 K4E42
ff99SBnmr igb7 E4K42
ff99SBnmr igb7 K4E42
ff99SBnmr igb8 E4K42
ff99SBnmr igb8 K4E42
ff03.r1 igb1 E4K42
ff03.r1 igb1 K4E42
ff03.r1 igb2 E4K42
ff03.r1 igb2 K4E42
ff03.r1 igb5 E4K42
ff03.r1 igb5 K4E42
ff03.r1 igb7 E4K42
ff03.r1 igb7 K4E42
ff03.r1 igb8 E4K42
ff03.r1 igb8 K4E42
ff14SB igb1 E4K42
ff14SB igb1 K4E42
ff14SB igb2 E4K42
ff14SB igb2 K4E42
ff14SB igb5 E4K42
ff14SB igb5 K4E42
ff14SB igb7 E4K42
ff14SB igb7 K4E42
ff14SB igb8 E4K42
ff14SB igb8 K4E42
Helicity (%)
Time (μs)
time (μs)

Figure S5. Percentage α-helicity of the $(E_4K_4)_2$ and $(K_4E_4)_2$ peptides for 27 forcefield–GB model combinations. The results are presented as percentage helicity as a function of simulation time. For each subplot, the dots represent the actual calculated values, and the solid line corresponds to the moving average over a window of 50 ns. The plots are colored by GB model-forcefield combination.

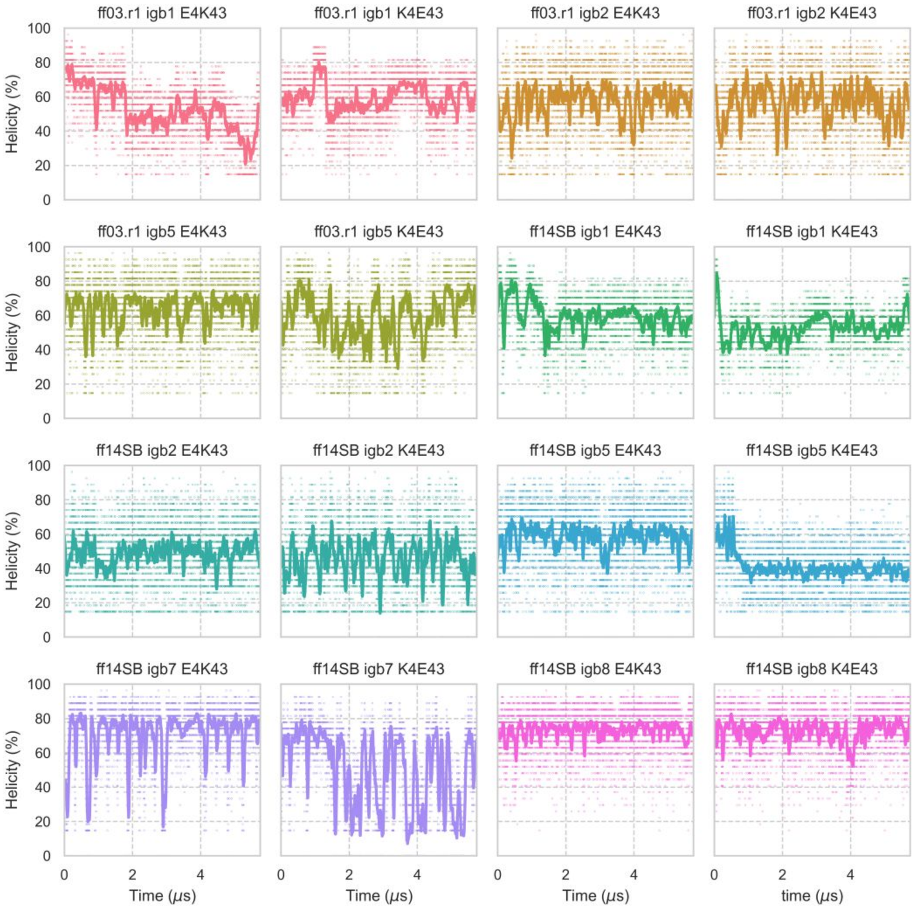

Figure S6. Percentage helicity of the $(E_4K_4)_3$ and $(K_4E_4)_3$ peptides for 8 forcefield–GB model combinations. The results are presented as percentage helicity as a function of simulation time. For each subplot, the dots represent the actual calculated values, and the solid line corresponds to the moving average over a window of 50 ns. The plots are colored by GB model-forcefield combination.

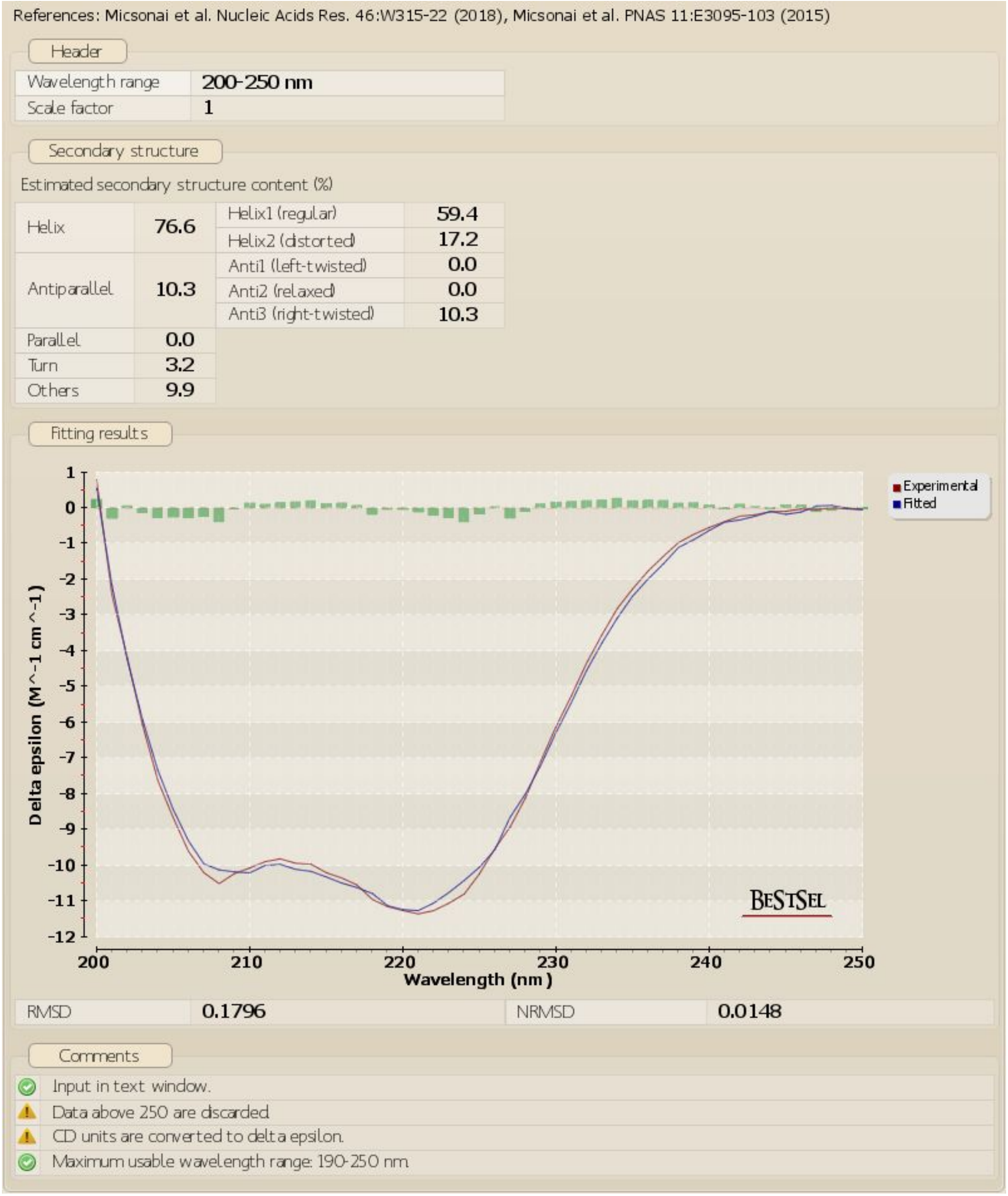

Figure S7. Screenshot showing the results of the BeStSel fitting of the experimental CD curve for $A_4(K_4E_4)_1A_4(K_4E_4)_1A_4$ in the 200-250 nm region, including the estimated secondary structure contents. In the plot, the red line corresponds to the experimental data, the blue line to the fit and the green bars to the residuals.

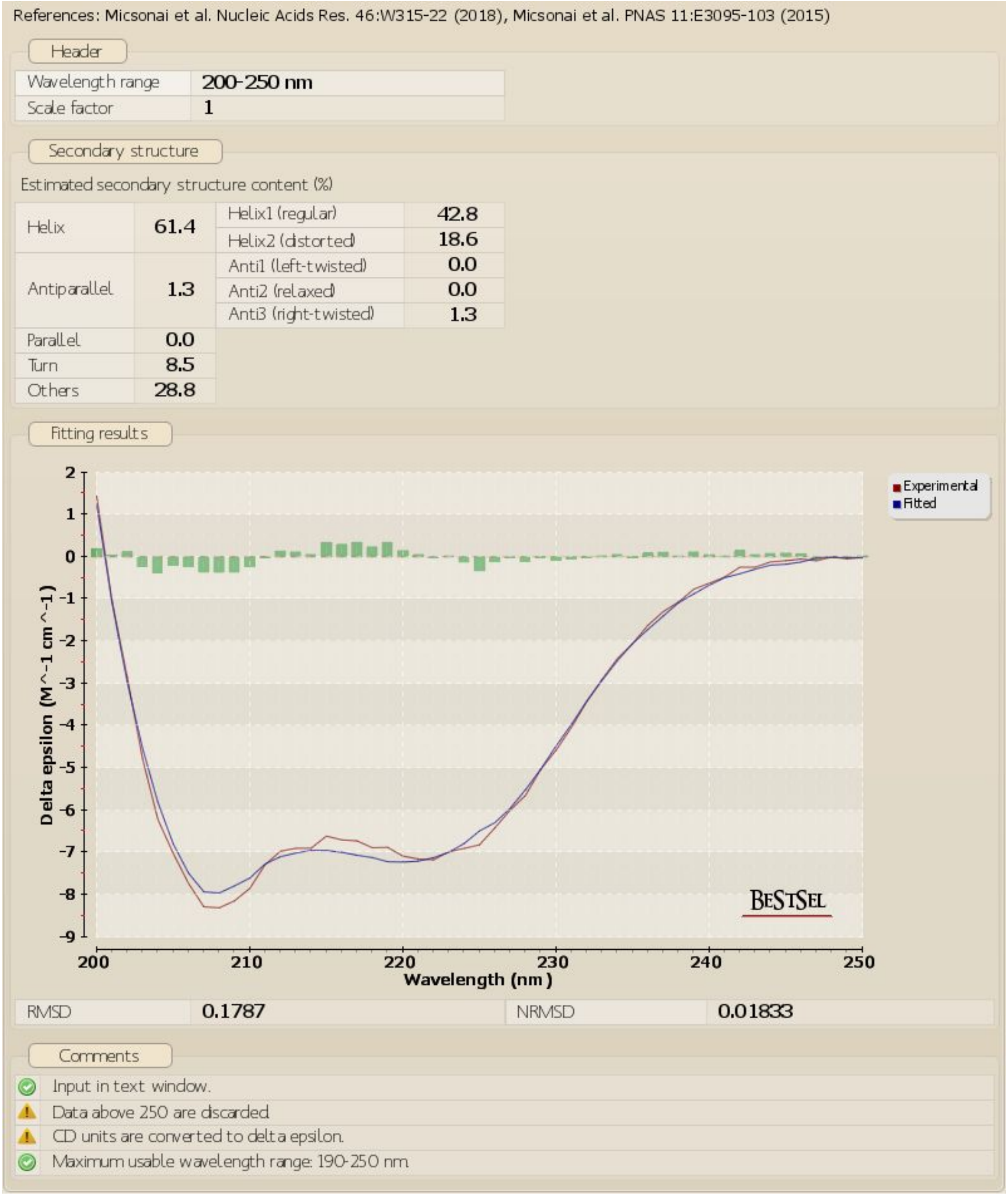

Figure S8. Screenshot showing the results of the BeStSel fitting of the experimental CD curve for $(E_4K_4)_2$ in the 200-250 nm region, including the estimated secondary structure contents. In the plot, the red line corresponds to the experimental data, the blue line to the fit and the green bars to the residuals.

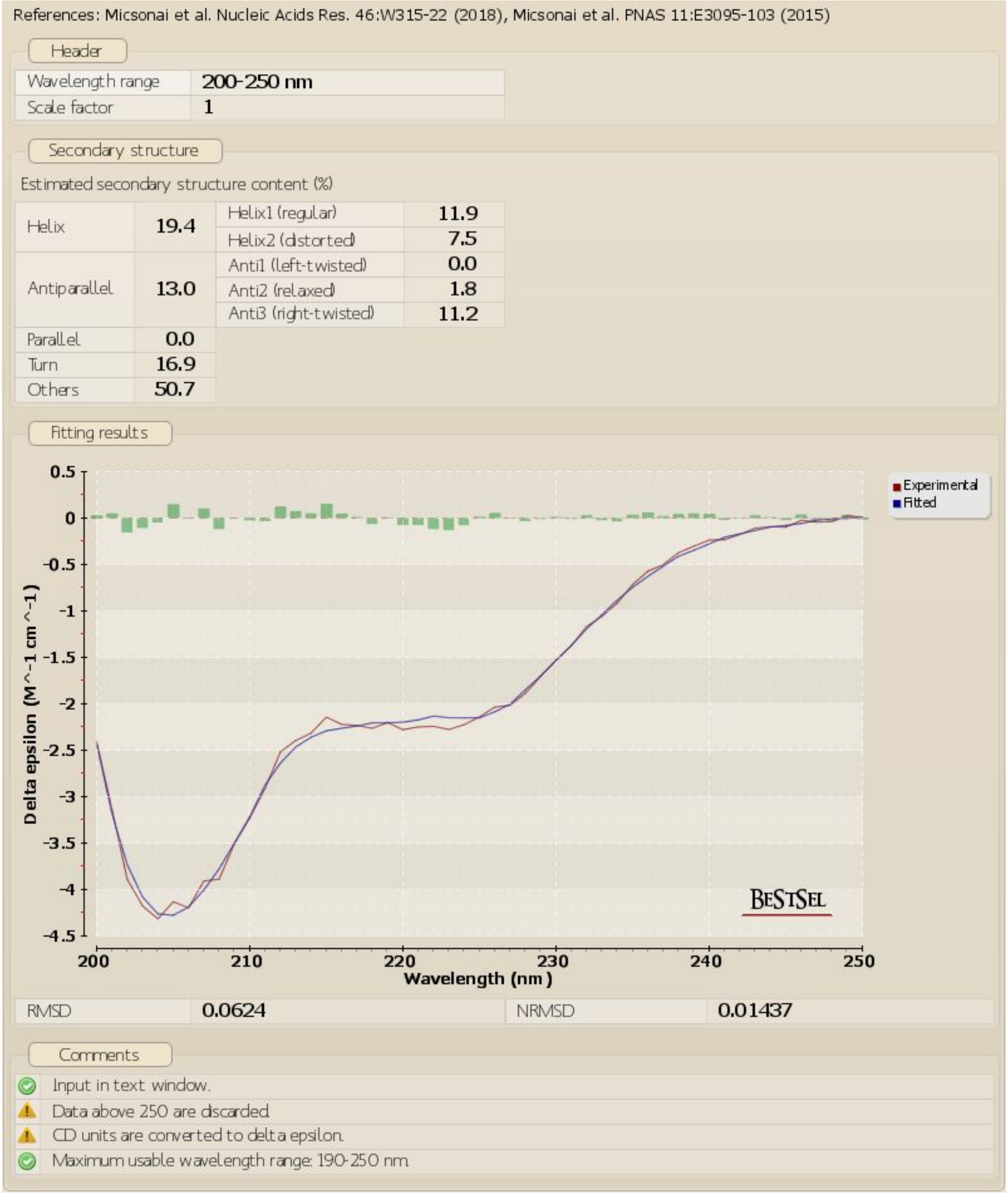

Figure S9. Screenshot showing the results of the BeStSel fitting of the experimental CD curve for $(K_4E_4)_2$ in the 200-250 nm region, including the estimated secondary structure contents. In the

plot, the red line corresponds to the experimental data, the blue line to the fit and the green bars to the residuals.

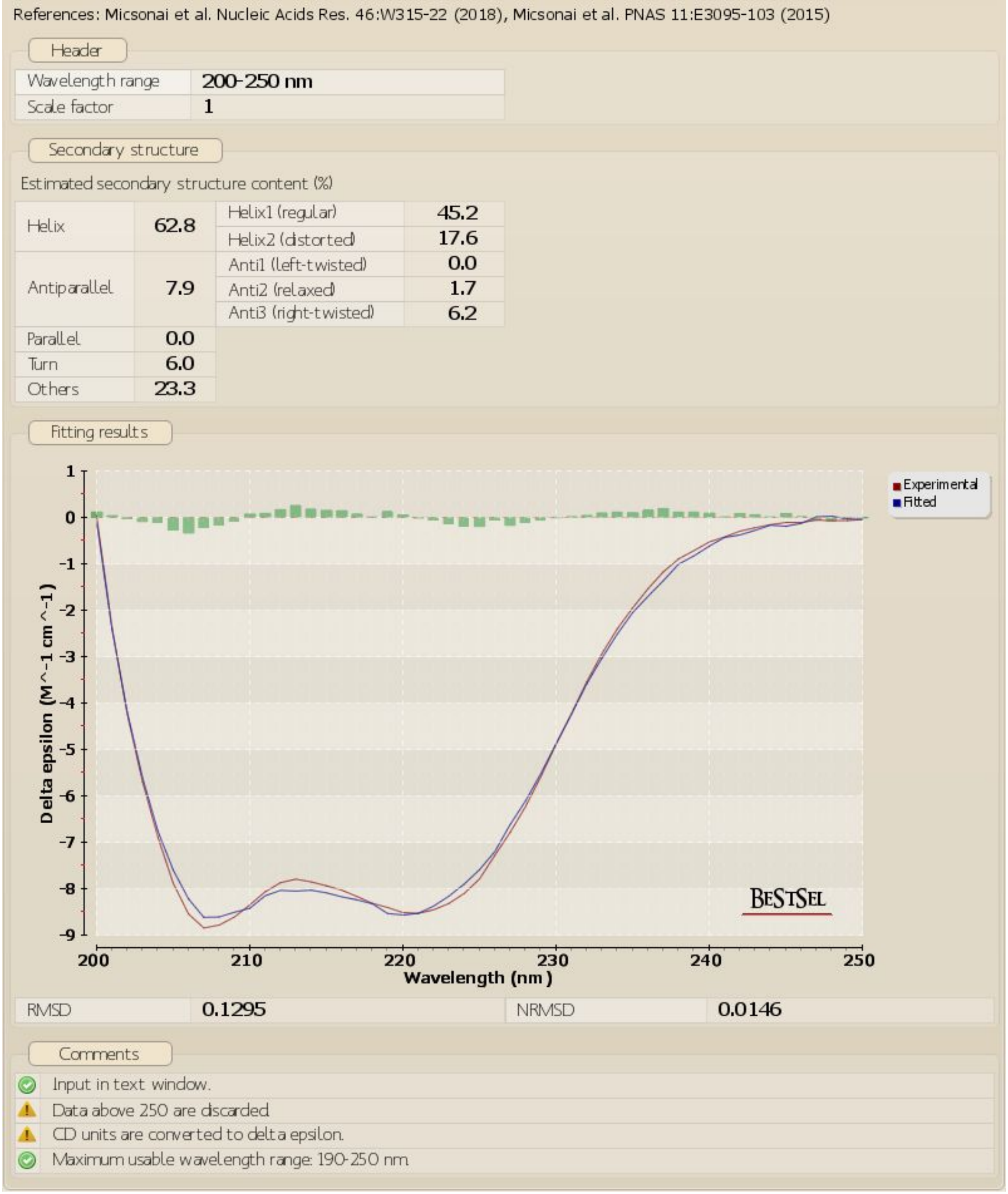

Figure S10. Screenshot showing the results of the BeStSel fitting of the experimental CD curve for $(E_4K_4)_3$ in the 200-250 nm region, including the estimated secondary structure contents. In the

plot, the red line corresponds to the experimental data, the blue line to the fit and the green bars to the residuals.

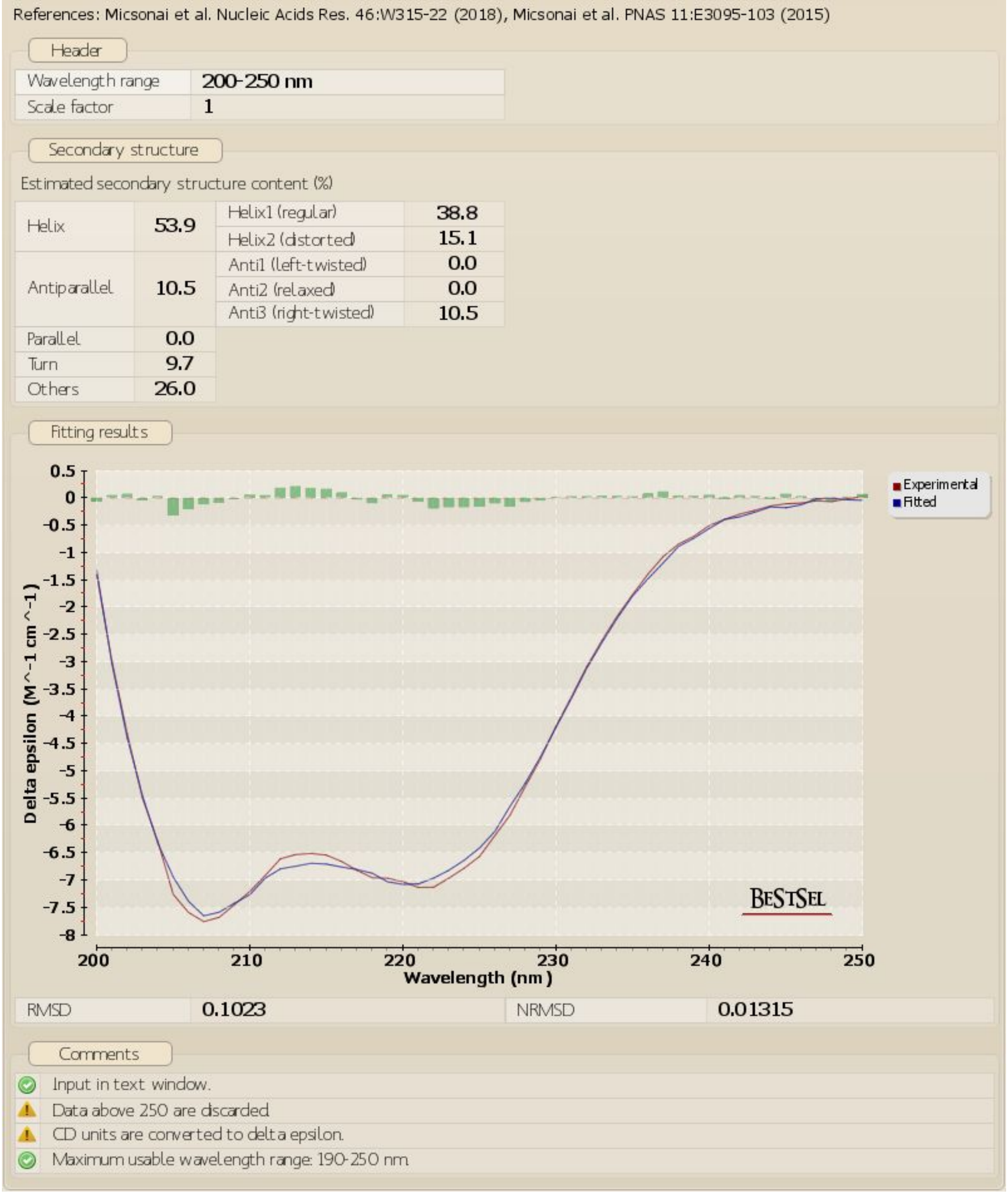

Figure S11. Screenshot showing the results of the BeStSel fitting of the experimental CD curve for $(K_4E_4)_3$ in the 200-250 nm region, including the estimated secondary structure contents. In the

plot, the red line corresponds to the experimental data, the blue line to the fit and the green bars to the residuals.

SUPPLEMENTARY TABLE

Table S1. Fraction helicity calculated from experimental CD data using BeStSel

		BeStSel fit of CD spectra[b]				
Peptide[a]	α-helicity using CD MRE_{222} (%)[22a]	α-helix (%)	Antiparallel β-sheet (%)	Parallel β-sheet (%)	Turn (%)	Others (%)[c]
$A_4(K_4E_4)_1A_4(K_4E_4)_1A_4$	97	77	10	0	3	10
$(E_4K_4)_2$	65	61	1	0	9	29
$(K_4E_4)_2$	22	19	13	0	17	51
$(E_4K_4)_3$	74	63	8	0	6	23
$(K_4E_4)_3$	62	54	10	0	10	26

[a]Method used in[22]: $Fraction\ helix = \left[\frac{(MRE_{222} - [\theta]_{coil})}{([\theta]_{helix} - [\theta]_{coil})}\right]$ with $[\theta]_{coil} = 640 - 45.T$ where T is the temperature in °C and $[\theta]_{helix} = -42{,}000 \times (1 - (3/n))$ where n is the number of peptide bonds (including from capping)[28]. [b]Obtained form BeStSel fitting in the 200 – 250 nm region. [c]includes coil, bend, β bridge, π helix and 3_{10} helix.

SUPPLEMENTARY MOVIES

Movie S1. Trajectories of the 6 μs MD simulations of $A_4(K_4E_4)_1A_4(K_4E_4)_1A_4$ using igb5 with ff14SB (left), igb5 with ff99SBnmr (center) and igb8 with ff14SBonlysc (right). The structures are colored by secondary structures: α-helix in blue, extended β-strand and β-bridge in green, π-helix in red, 3_{10} helix in purple, turn in orange and coil in white. Igb5 with ff99SBnmr (center) simulated the highest median % α-helix of 87%. The most recent GB model–forcefield combination, igb8 with ff14SBonlysc, did not maintain α-helicity (median = 26%), whereas igb5 with ff14SB, (which was actually parameterized for use with TIP3P water), led to a higher degree of helicity, 71%.

Movie S2. Trajectories of the 6 μs MD simulations of $A_4(K_4E_4)_1A_4(K_4E_4)_1A_4$ using ff96 in combination with igb5 (left), igb7 (center) and igb8 (right). The structures are colored by secondary structures: α-helix in blue, extended β-strand and β-bridge in green, π-helix in red, 3_{10} helix in purple, turn in orange and coil in white. Igb5 with ff96 yield a mostly α-helical structure

(median = 83%), in agreement with experimental data (97%)[22] whereas igb7 with ff96 led to a disordered structure and igb8 with ff96 to a stable β-sheet.

REFERENCES

(1) Case, D. A.; Walker, R. C.; Cheatham, T. E.; Simmerling, C.; Roitberg, A. E.; Merz, K. M.; Luo, R.; Darden, T.; Wang, J.; Duke, R. E.; Le Grand, S.; Swails, J. M.; Cerutti, D. S.; Monard, G.; Sagui, C.; Kaus, J.; Betz, R.; Madej, B.; Lin, C.; Mermelstein, D.; Li, P.; Onufriev, A. V.; Izadi, S.; Wolf, R. M.; Wu, X.; Götz, A. W.; Gohlke, H.; Homeyer, N.; Botello-Smith, W. M.; Xiao, L.; Luchko, T.; Giese, T.; Lee, T.; Nguyen, H. T.; Nguyen, H.; Janowski, P.; Omelyan, I.; Kovalenko, A.; Kollman, P. A. *Amber 2016*; 2016.

(2) Pettersen, E. F.; Goddard, T. D.; Huang, C. C.; Couch, G. S.; Greenblatt, D. M.; Meng, E. C.; Ferrin, T. E. UCSF Chimera—A Visualization System for Exploratory Research and Analysis. *J. Comput. Chem.* **2004**, *25* (13), 1605–1612. https://doi.org/10.1002/jcc.20084.

(3) Shapovalov, M. V.; Dunbrack, R. L. A Smoothed Backbone-Dependent Rotamer Library for Proteins Derived from Adaptive Kernel Density Estimates and Regressions. *Structure* **2011**, *19* (6), 844–858. https://doi.org/10.1016/j.str.2011.03.019.

(4) Hawkins, G. D.; Cramer, C. J.; Truhlar, D. G. Pairwise Solute Descreening of Solute Charges from a Dielectric Medium. *Chem. Phys. Lett.* **1995**, *246* (1), 122–129. https://doi.org/10.1016/0009-2614(95)01082-K.

(5) Onufriev, A.; Bashford, D.; Case, D. A. Modification of the Generalized Born Model Suitable for Macromolecules. *J. Phys. Chem. B* **2000**, *104* (15), 3712–3720. https://doi.org/10.1021/jp994072s.

(6) Onufriev, A.; Bashford, D.; Case, D. A. Exploring Protein Native States and Large-Scale Conformational Changes with a Modified Generalized Born Model. *Proteins Struct. Funct. Bioinforma.* **2004**, *55* (2), 383–394. https://doi.org/10.1002/prot.20033.

(7) Mongan, J.; Simmerling, C.; McCammon, J. A.; Case, D. A.; Onufriev, A. Generalized Born Model with a Simple, Robust Molecular Volume Correction. *J. Chem. Theory Comput.* **2007**, *3* (1), 156–169. https://doi.org/10.1021/ct600085e.

(8) Nguyen, H.; Roe, D. R.; Simmerling, C. Improved Generalized Born Solvent Model Parameters for Protein Simulations. *J. Chem. Theory Comput.* **2013**, *9* (4), 2020–2034. https://doi.org/10.1021/ct3010485.

(9) Cornell, W. D.; Cieplak, P.; Bayly, C. I.; Gould, I. R.; Merz, K. M.; Ferguson, D. M.; Spellmeyer, D. C.; Fox, T.; Caldwell, J. W.; Kollman, P. A. A Second Generation Force Field for the Simulation of Proteins, Nucleic Acids, and Organic Molecules. *J. Am. Chem. Soc.* **1995**, *117* (19), 5179–5197. https://doi.org/10.1021/ja00124a002.

(10) Kollman, P.; Dixon, R.; Cornell, W.; Fox, T.; Chipot, C.; Pohorille, A. The Development/Application of a 'Minimalist' Organic/Biochemical Molecular Mechanic Force Field Using a Combination of Ab Initio Calculations and Experimental Data. In *Computer Simulation of Biomolecular Systems: Theoretical and Experimental Applications*; van Gunsteren, W. F., Weiner, P. K., Wilkinson, A. J., Eds.; Computer Simulations of Biomolecular Systems; Springer Netherlands: Dordrecht, 1997; pp 83–96. https://doi.org/10.1007/978-94-017-1120-3_2.

(11) III, T. E. C.; Cieplak, P.; Kollman, P. A. A Modified Version of the Cornell et al. Force Field with Improved Sugar Pucker Phases and Helical Repeat. *J. Biomol. Struct. Dyn.* **1999**, *16* (4), 845–862. https://doi.org/10.1080/07391102.1999.10508297.

(12) Wang, J.; Cieplak, P.; Kollman, P. A. How well does a restrained electrostatic potential (RESP) model perform in calculating conformational energies of organic and biological molecules? *J. Comput. Chem.* **2000**, *21* (12), 1049–1074. https://doi.org/10.1002/1096-987X(200009)21:12<1049::AID-JCC3>3.0.CO;2-F.

(13) Hornak, V.; Abel, R.; Okur, A.; Strockbine, B.; Roitberg, A.; Simmerling, C. Comparison of Multiple Amber Force Fields and Development of Improved Protein Backbone Parameters. *Proteins Struct. Funct. Bioinforma.* **2006**, *65* (3), 712–725. https://doi.org/10.1002/prot.21123.

(14) Lindorff-Larsen, K.; Piana, S.; Palmo, K.; Maragakis, P.; Klepeis, J. L.; Dror, R. O.; Shaw, D. E. Improved Side-Chain Torsion Potentials for the Amber Ff99SB Protein Force Field. *Proteins Struct. Funct. Bioinforma.* **2010**, *78* (8), 1950–1958. https://doi.org/10.1002/prot.22711.

(15) Li, D.-W.; Brüschweiler, R. NMR-Based Protein Potentials. *Angew. Chem. Int. Ed.* **2010**, *49* (38), 6778–6780. https://doi.org/10.1002/anie.201001898.

(16) Duan, Y.; Wu, C.; Chowdhury, S.; Lee, M. C.; Xiong, G.; Zhang, W.; Yang, R.; Cieplak, P.; Luo, R.; Lee, T.; Caldwell, J.; Wang, J.; Kollman, P. A point-charge force field for molecular mechanics simulations of proteins based on condensed-phase quantum mechanical calculations. *J. Comput. Chem.* **2003**, *24* (16), 1999–2012. https://doi.org/10.1002/jcc.10349.

(17) Maier, J. A.; Martinez, C.; Kasavajhala, K.; Wickstrom, L.; Hauser, K. E.; Simmerling, C. Ff14SB: Improving the Accuracy of Protein Side Chain and Backbone Parameters from Ff99SB. *J. Chem. Theory Comput.* **2015**, *11* (8), 3696–3713. https://doi.org/10.1021/acs.jctc.5b00255.

(18) Cerutti, D. S.; Swope, W. C.; Rice, J. E.; Case, D. A. Ff14ipq: A Self-Consistent Force Field for Condensed-Phase Simulations of Proteins. *J. Chem. Theory Comput.* **2014**, *10* (10), 4515–4534. https://doi.org/10.1021/ct500643c.

(19) Wang, L.-P.; McKiernan, K. A.; Gomes, J.; Beauchamp, K. A.; Head-Gordon, T.; Rice, J. E.; Swope, W. C.; Martínez, T. J.; Pande, V. S. Building a More Predictive Protein Force Field: A Systematic and Reproducible Route to AMBER-FB15. *J. Phys. Chem. B* **2017**, *121* (16), 4023–4039. https://doi.org/10.1021/acs.jpcb.7b02320.

(20) Debiec, K. T.; Cerutti, D. S.; Baker, L. R.; Gronenborn, A. M.; Case, D. A.; Chong, L. T. Further along the Road Less Traveled: AMBER Ff15ipq, an Original Protein Force Field Built on a Self-Consistent Physical Model. *J. Chem. Theory Comput.* **2016**, *12* (8), 3926–3947. https://doi.org/10.1021/acs.jctc.6b00567.

(21) Hopkins, C. W.; Le Grand, S.; Walker, R. C.; Roitberg, A. E. Long-Time-Step Molecular Dynamics through Hydrogen Mass Repartitioning. *J. Chem. Theory Comput.* **2015**, *11* (4), 1864–1874. https://doi.org/10.1021/ct5010406.

(22) Baker, E. G.; Bartlett, G. J.; Crump, M. P.; Sessions, R. B.; Linden, N.; Faul, C. F. J.; Woolfson, D. N. Local and Macroscopic Electrostatic Interactions in Single α-Helices. *Nat. Chem. Biol.* **2015**, *11* (3), 221–228. https://doi.org/10.1038/nchembio.1739.

(23) Götz, A. W.; Williamson, M. J.; Xu, D.; Poole, D.; Le Grand, S.; Walker, R. C. Routine Microsecond Molecular Dynamics Simulations with AMBER on GPUs. 1. Generalized Born. *J. Chem. Theory Comput.* **2012**, *8* (5), 1542–1555. https://doi.org/10.1021/ct200909j.

(24) Kabsch, W.; Sander, C. Dictionary of Protein Secondary Structure: Pattern Recognition of Hydrogen-Bonded and Geometrical Features. *Biopolymers* **1983**, *22* (12), 2577–2637. https://doi.org/10.1002/bip.360221211.

(25) Roe, D. R.; Cheatham, T. E. PTRAJ and CPPTRAJ: Software for Processing and Analysis of Molecular Dynamics Trajectory Data. *J. Chem. Theory Comput.* **2013**, *9* (7), 3084–3095. https://doi.org/10.1021/ct400341p.

(26) Micsonai, A.; Wien, F.; Bulyáki, É.; Kun, J.; Moussong, É.; Lee, Y.-H.; Goto, Y.; Réfrégiers, M.; Kardos, J. BeStSel: A Web Server for Accurate Protein Secondary Structure Prediction and Fold Recognition from the Circular Dichroism Spectra. *Nucleic Acids Res.* **2018**, *46* (W1), W315–W322. https://doi.org/10.1093/nar/gky497.

(27) Micsonai, A.; Wien, F.; Kernya, L.; Lee, Y.-H.; Goto, Y.; Réfrégiers, M.; Kardos, J. Accurate Secondary Structure Prediction and Fold Recognition for Circular Dichroism Spectroscopy. *Proc. Natl. Acad. Sci.* **2015**, *112* (24), E3095–E3103. https://doi.org/10.1073/pnas.1500851112.

(28) Scholtz, J. M.; Qian, H.; York, E. J.; Stewart, J. M.; Baldwin, R. L. Parameters of Helix–Coil Transition Theory for Alanine-Based Peptides of Varying Chain Lengths in Water. *Biopolymers* **1991**, *31* (13), 1463–1470. https://doi.org/10.1002/bip.360311304.
